# Supplementary material for: VCP inhibition induces an unfolded protein response and apoptosis in human acute myeloid leukemia cells
Source: PLoS One. 2022 Apr 6;17(4):e0266478. doi: 10.1371/journal.pone.0266478 (PMC8986003; doi:10.1371/journal.pone.0266478)
Supplement: S1 Raw images — (PDF) [file pone.0266478.s002.pdf]

Raw images corresponding to Fig. 1a

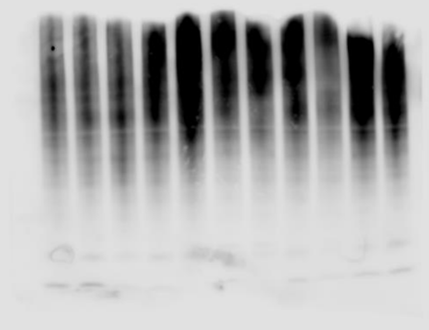

Primary antibody:
Ubiquitin

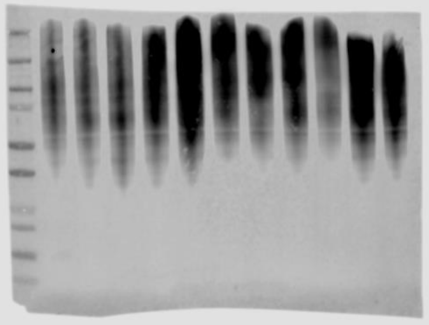

Primary antibody:
Ubiquitin

Lanes (left to right):
Protein marker (10, 15, 20, 25, 37, 50, 75, 100, 150, 250 kDa)
THP-1 / NT
THP-1 / DMSO
THP-1 / MG-132 (500 nM)
THP-1 / MG-132 (5 µM)
THP-1 / MG-132 (50 µM)
THP-1 / CB-5083 (500 nM)
THP-1 / CB-5083 (5 µM)
THP-1 / CB-5083 (50 µM)
THP-1 / NMS-873 (500 nM)
THP-1 / NMS-873 (5 µM)
THP-1 / NMS-873 (50 µM)

Raw images corresponding to Fig. 1a

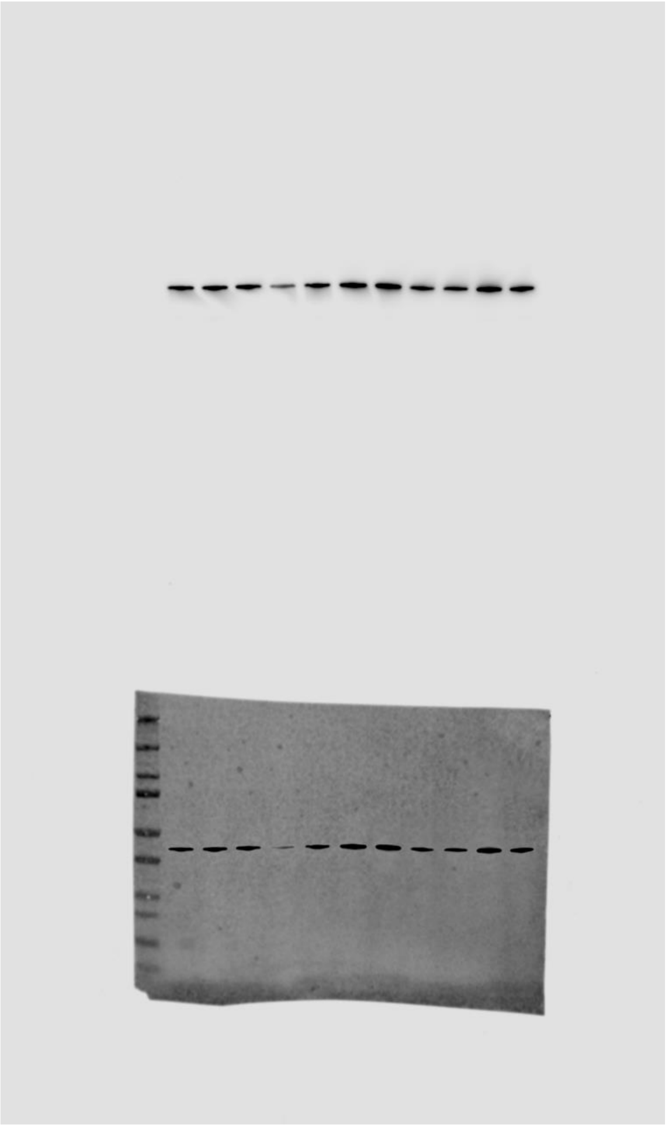

Primary antibody:
β-actin

Primary antibody:
β-actin

Lanes (left to right):
Protein marker (10, 15, 20, 25, 37, 50, 75, 100, 150, 250 kDa)
THP-1 / NT
THP-1 / DMSO
THP-1 / MG-132 (500 nM)
THP-1 / MG-132 (5 µM)
THP-1 / MG-132 (50 µM)
THP-1 / CB-5083 (500 nM)
THP-1 / CB-5083 (5 µM)
THP-1 / CB-5083 (50 µM)
THP-1 / NMS-873 (500 nM)
THP-1 / NMS-873 (5 µM)
THP-1 / NMS-873 (50 µM)

Raw images corresponding to Fig. 1b

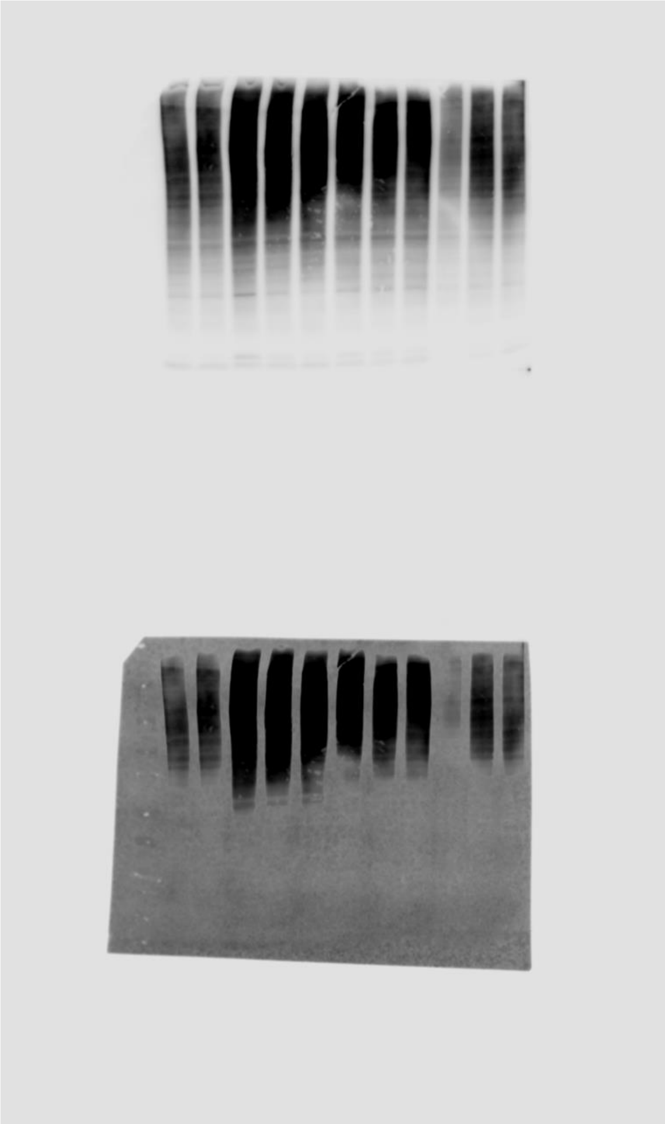

Primary antibody:
Ubiquitin

Primary antibody:
Ubiquitin

Lanes (left to right):
Protein marker (10, 15, 20, 25, 37, 50, 75, 100, 150, 250 kDa)
FFM05 / NT
FFM05 / DMSO
FFM05 / MG-132 (500 nM)
FFM05 / MG-132 (5 µM)
FFM05 / MG-132 (50 µM)
FFM05 / CB-5083 (500 nM)
FFM05 / CB-5083 (5 µM)
FFM05 / CB-5083 (50 µM)
FFM05 / NMS-873 (500 nM)
FFM05 / NMS-873 (5 µM)
FFM05 / NMS-873 (50 µM)

Raw images corresponding to Fig. 1b

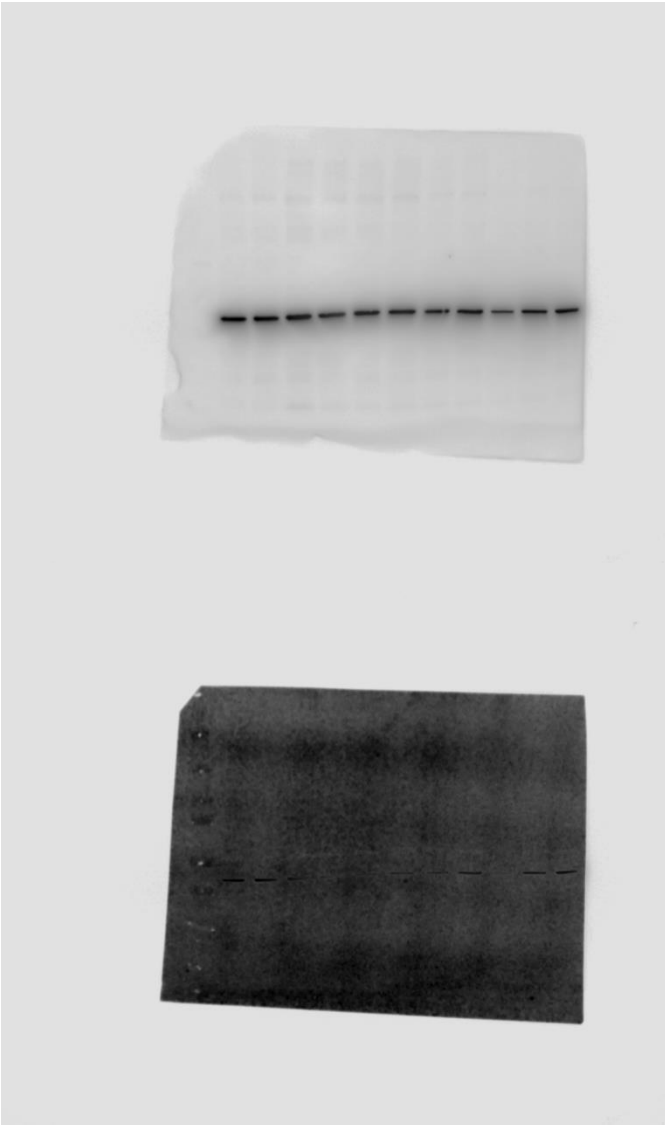

Primary antibody:
β-actin

Primary antibody:
β-actin

Lanes (left to right):
Protein marker (10, 15, 20, 25, 37, 50, 75, 100, 150, 250 kDa)
FFM05 / NT
FFM05 / DMSO
FFM05 / MG-132 (500 nM)
FFM05 / MG-132 (5 µM)
FFM05 / MG-132 (50 µM)
FFM05 / CB-5083 (500 nM)
FFM05 / CB-5083 (5 µM)
FFM05 / CB-5083 (50 µM)
FFM05 / NMS-873 (500 nM)
FFM05 / NMS-873 (5 µM)
FFM05 / NMS-873 (50 µM)

Raw images corresponding to Fig. 1c

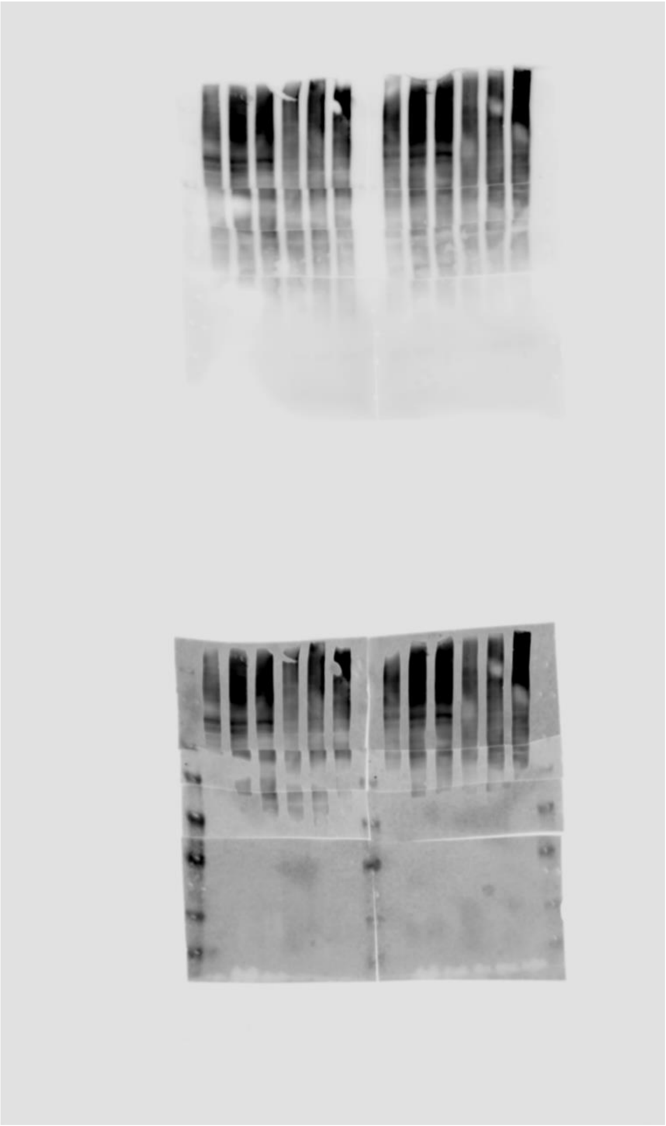

Primary antibody:
Ubiquitin

Primary antibody:
Ubiquitin

Lanes (left to right):
Protein marker (6, 14, 17, 28, 38, 49, 62, 98, 198 kDa)
MV4-11 / NT
MV4-11 / CB-5083 (200 nM)
MV4-11 / CB-5083 (500 nM)
THP-1 / NT
THP-1 / CB-5083 (200 nM)
THP-1 / CB-5083 (500 nM)
Protein marker (6, 14, 17, 28, 38, 49, 62, 98, 198 kDa)
MV4-11 / NT
MV4-11 / CB-5083 (200 nM)
MV4-11 / CB-5083 (500 nM)
THP-1 / NT
THP-1 / CB-5083 (200 nM)
THP-1 / CB-5083 (500 nM)
Protein marker (6, 14, 17, 28, 38, 49, 62, 98, 198 kDa)

Raw images corresponding to Fig. 1c

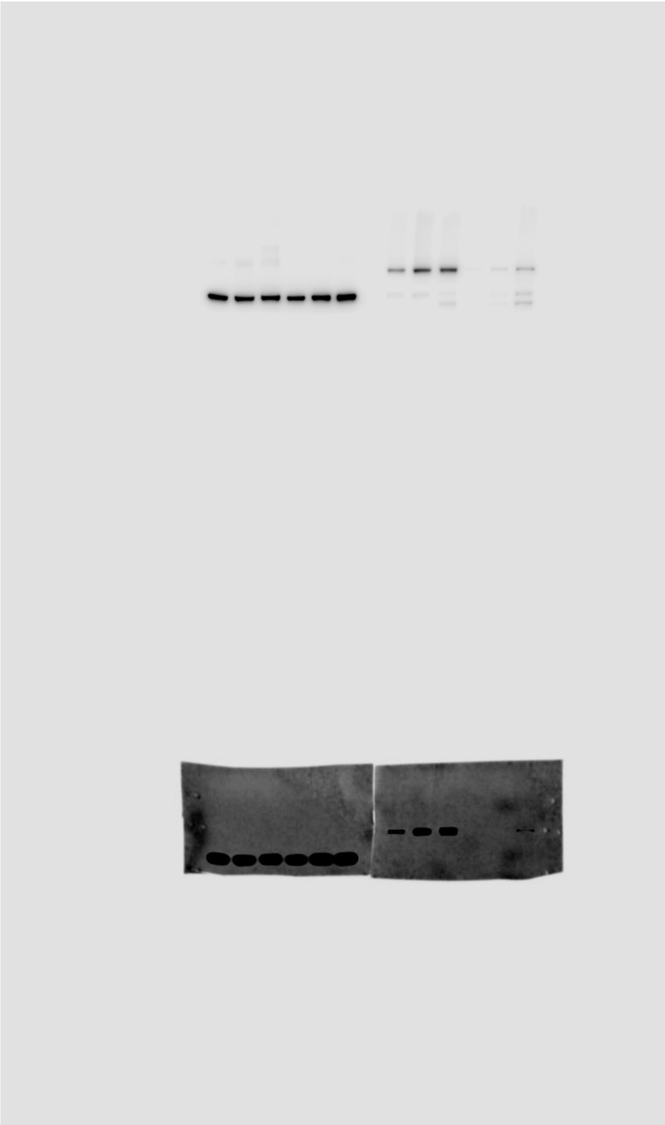

Primary antibody:
Calnexin / IRE1α

Primary antibody:
Calnexin / IRE1α

Lanes (left to right):
Protein marker (6, 14, 17, 28, 38, 49, 62, 98, 198 kDa)
MV4-11 / NT
MV4-11 / CB-5083 (200 nM)
MV4-11 / CB-5083 (500 nM)
THP-1 / NT
THP-1 / CB-5083 (200 nM)
THP-1 / CB-5083 (500 nM)
Protein marker (6, 14, 17, 28, 38, 49, 62, 98, 198 kDa)
MV4-11 / NT
MV4-11 / CB-5083 (200 nM)
MV4-11 / CB-5083 (500 nM)
THP-1 / NT
THP-1 / CB-5083 (200 nM)
THP-1 / CB-5083 (500 nM)
Protein marker (6, 14, 17, 28, 38, 49, 62, 98, 198 kDa)

Raw images corresponding to Fig. 1c

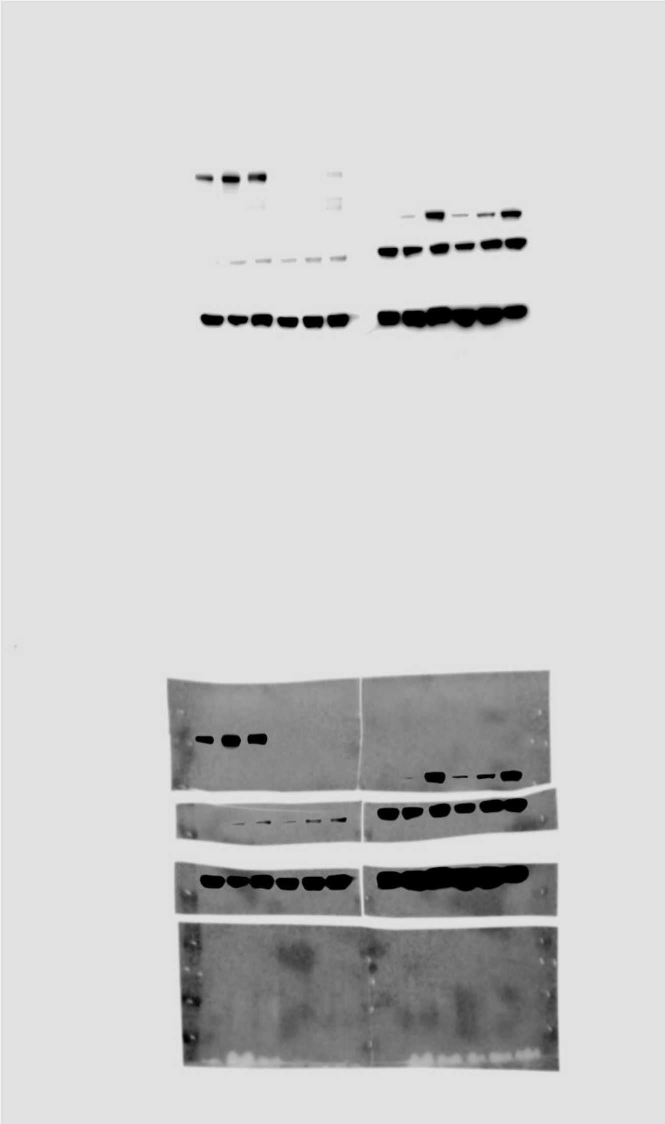

Primary antibody:
PERK / BiP

Primary antibody:
Ero1-α / PDI

Primary antibody:
β-actin

Primary antibody:
CHOP

Primary antibody:
PERK / BiP

Primary antibody:
Ero1-α / PDI

Primary antibody:
β-actin

Primary antibody:
CHOP

Lanes (left to right):
Protein marker (6, 14, 17, 28, 38, 49, 62, 98, 198 kDa)
MV4-11 / NT
MV4-11 / CB-5083 (200 nM)
MV4-11 / CB-5083 (500 nM)
THP-1 / NT
THP-1 / CB-5083 (200 nM)
THP-1 / CB-5083 (500 nM)
Protein marker (6, 14, 17, 28, 38, 49, 62, 98, 198 kDa)
MV4-11 / NT
MV4-11 / CB-5083 (200 nM)
MV4-11 / CB-5083 (500 nM)
THP-1 / NT
THP-1 / CB-5083 (200 nM)
THP-1 / CB-5083 (500 nM)
Protein marker (6, 14, 17, 28, 38, 49, 62, 98, 198 kDa)

Raw images corresponding to Fig. 6a

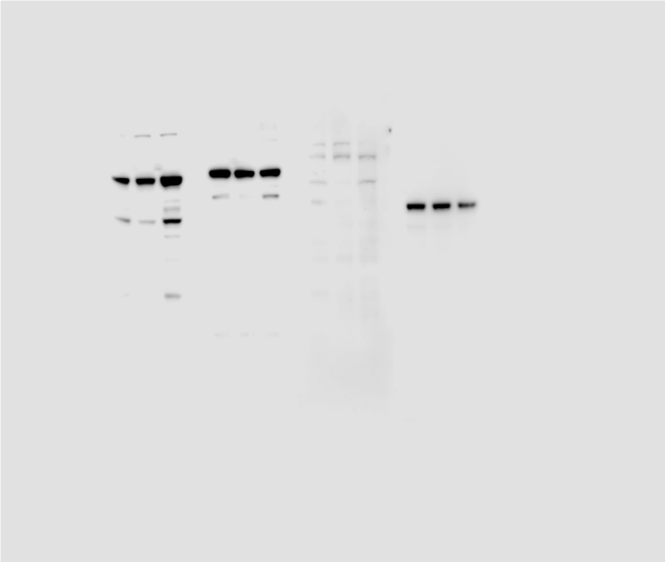

Primary antibody:
BiP / Calnexin /
CHOP / Ero1-α

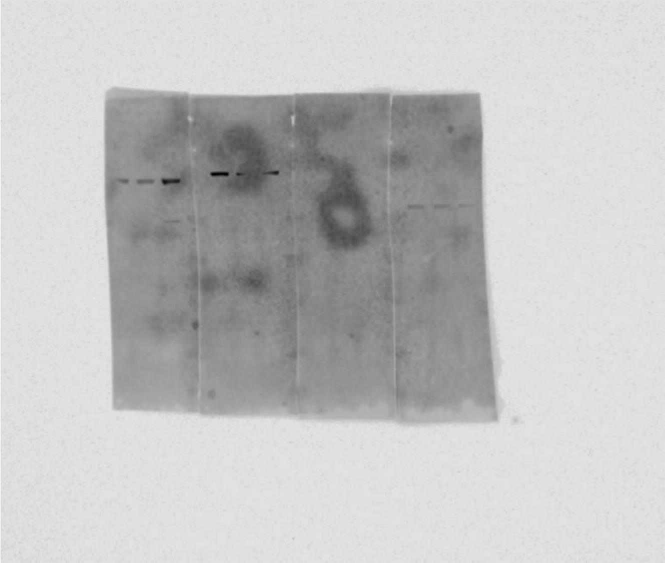

Primary antibody:
BiP / Calnexin /
CHOP / Ero1-α

Lanes (left to right):
MV4-11 / NT
MV4-11 / CB-5083 (200 nM)
MV4-11 / CB-5083 (500 nM)
Protein marker (6, 14, 17, 28, 38, 49, 62, 98, 198 kDa)
MV4-11 / NT
MV4-11 / CB-5083 (200 nM)
MV4-11 / CB-5083 (500 nM)
Protein marker (6, 14, 17, 28, 38, 49, 62, 98, 198 kDa)
MV4-11 / NT
MV4-11 / CB-5083 (200 nM)
MV4-11 / CB-5083 (500 nM)
Protein marker (6, 14, 17, 28, 38, 49, 62, 98, 198 kDa)
MV4-11 / NT
MV4-11 / CB-5083 (200 nM)
MV4-11 / CB-5083 (500 nM)

Raw images corresponding to Fig. 6a

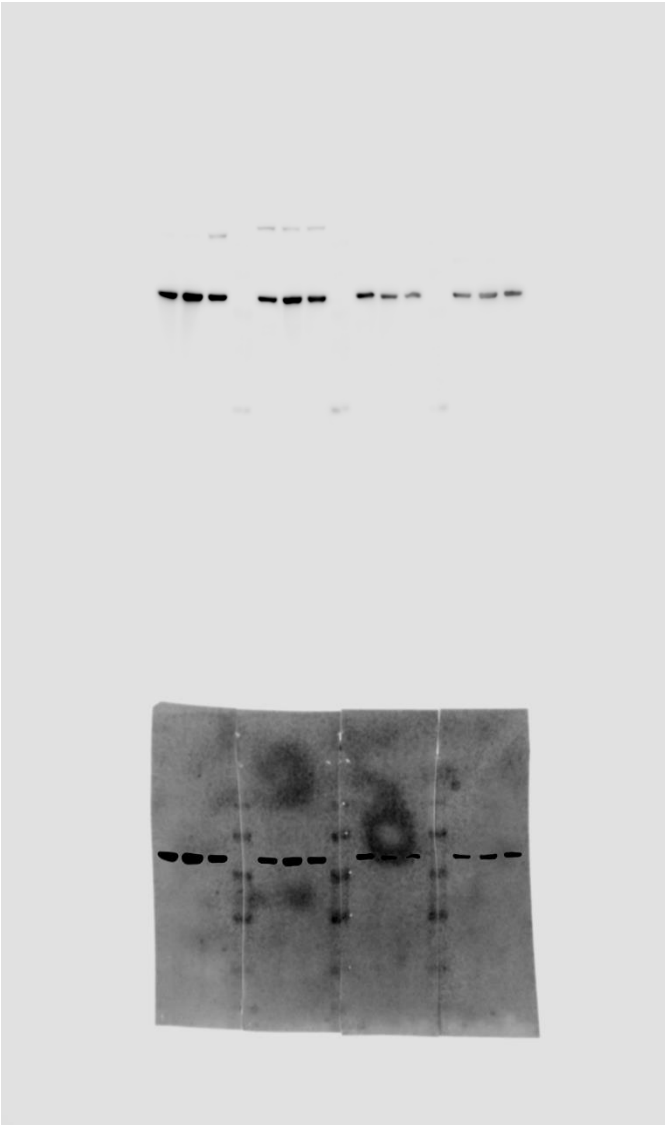

Primary antibody:
β-actin

Primary antibody:
β-actin

Lanes (left to right):
MV4-11 / NT
MV4-11 / CB-5083 (200 nM)
MV4-11 / CB-5083 (500 nM)
Protein marker (6, 14, 17, 28, 38, 49, 62, 98, 198 kDa)
MV4-11 / NT
MV4-11 / CB-5083 (200 nM)
MV4-11 / CB-5083 (500 nM)
Protein marker (6, 14, 17, 28, 38, 49, 62, 98, 198 kDa)
MV4-11 / NT
MV4-11 / CB-5083 (200 nM)
MV4-11 / CB-5083 (500 nM)
Protein marker (6, 14, 17, 28, 38, 49, 62, 98, 198 kDa)
MV4-11 / NT
MV4-11 / CB-5083 (200 nM)
MV4-11 / CB-5083 (500 nM)

Raw images corresponding to Fig. 6a

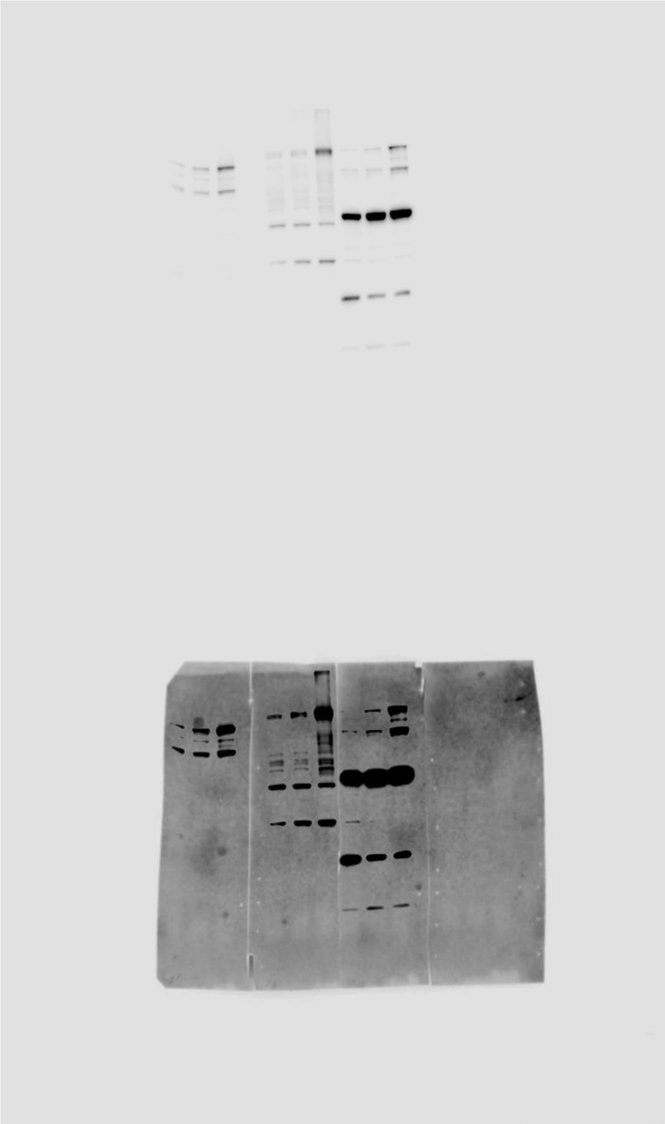

Primary antibody:
IRE1α / PERK / PDI

Primary antibody:
IRE1α / PERK / PDI

Lanes (left to right):
MV4-11 / NT
MV4-11 / CB-5083 (200 nM)
MV4-11 / CB-5083 (500 nM)
Protein marker (6, 14, 17, 28, 38, 49, 62, 98, 198 kDa)
MV4-11 / NT
MV4-11 / CB-5083 (200 nM)
MV4-11 / CB-5083 (500 nM)
MV4-11 / NT
MV4-11 / CB-5083 (200 nM)
MV4-11 / CB-5083 (500 nM)
Protein marker (6, 14, 17, 28, 38, 49, 62, 98, 198 kDa)
MV4-11 / NT
MV4-11 / CB-5083 (200 nM)
MV4-11 / CB-5083 (500 nM)
Protein marker (6, 14, 17, 28, 38, 49, 62, 98, 198 kDa)

Raw images corresponding to Fig. 6a

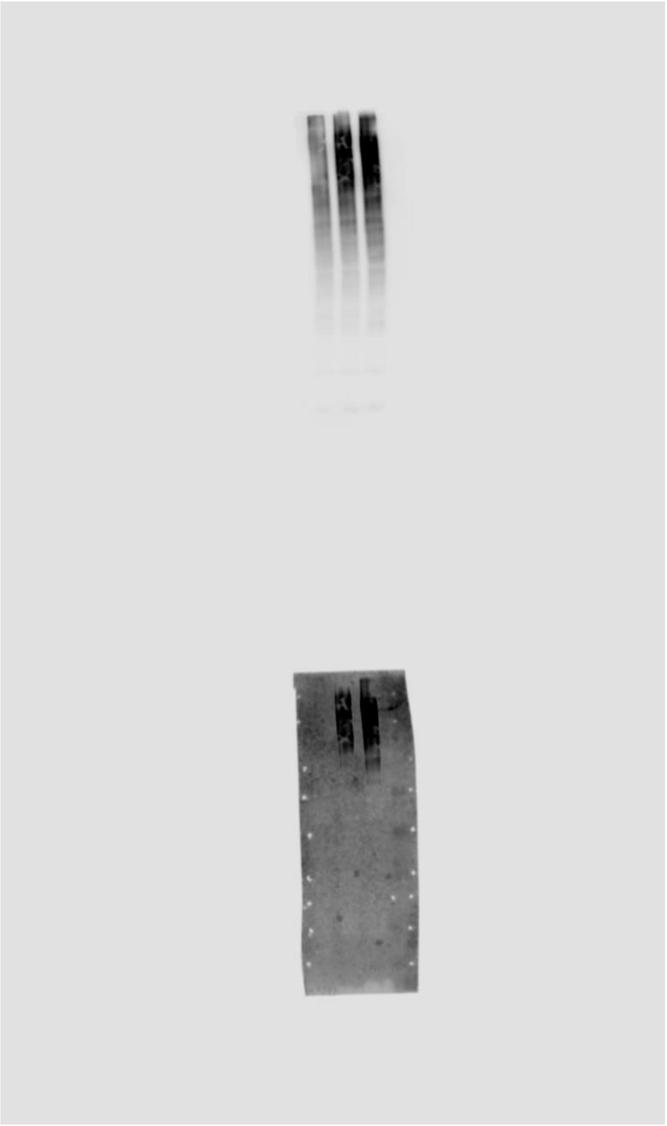

Primary antibody:
Ubiquitin

Primary antibody:
Ubiquitin

Lanes (left to right):
MV4-11 / NT
MV4-11 / CB-5083 (200 nM)
MV4-11 / CB-5083 (500 nM)
Protein marker (6, 14, 17, 28, 38, 49, 62, 98, 198 kDa)

Raw images corresponding to Fig. 6a

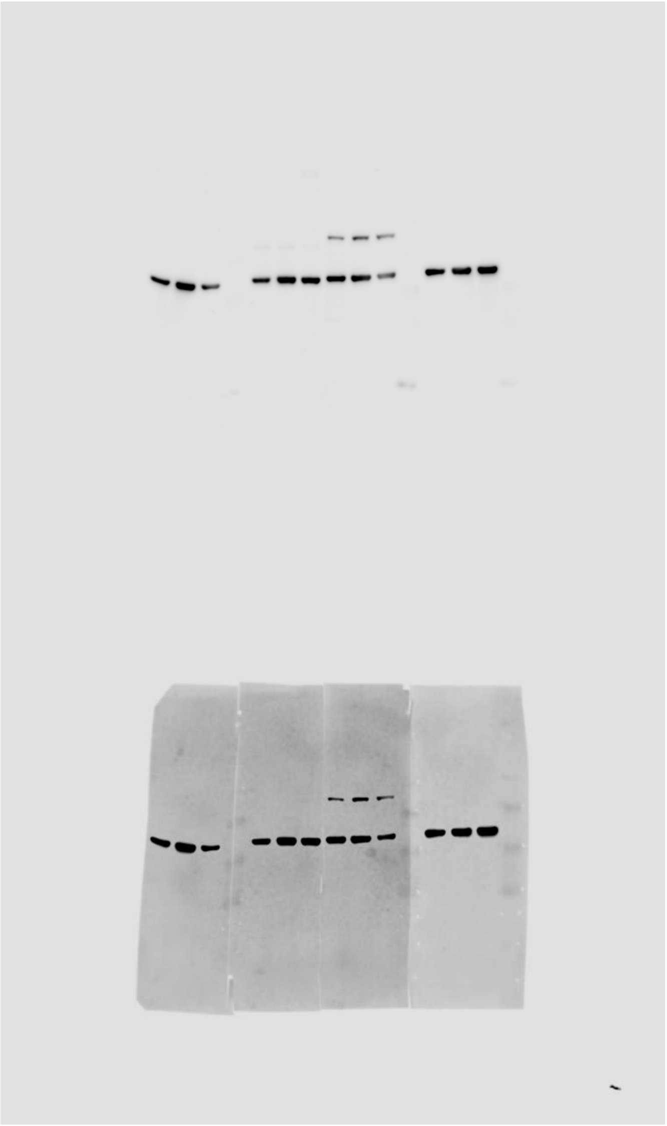

Primary antibody:
β-actin

Primary antibody:
β-actin

Lanes (left to right):
MV4-11 / NT
MV4-11 / CB-5083 (200 nM)
MV4-11 / CB-5083 (500 nM)
Protein marker (6, 14, 17, 28, 38, 49, 62, 98, 198 kDa)
MV4-11 / NT
MV4-11 / CB-5083 (200 nM)
MV4-11 / CB-5083 (500 nM)
MV4-11 / NT
MV4-11 / CB-5083 (200 nM)
MV4-11 / CB-5083 (500 nM)
Protein marker (6, 14, 17, 28, 38, 49, 62, 98, 198 kDa)
MV4-11 / NT
MV4-11 / CB-5083 (200 nM)
MV4-11 / CB-5083 (500 nM)
Protein marker (6, 14, 17, 28, 38, 49, 62, 98, 198 kDa)
